# Importance of Suitable Reference Gene Selection for Quantitative RT-PCR during ATDC5 Cells Chondrocyte Differentiation

**DOI:** 10.1371/journal.pone.0064786

**Published:** 2013-05-21

**Authors:** Zhichen Zhai, Yongchang Yao, Yingjun Wang

**Affiliations:** 1 School of Materials Science and Engineering, South China University of Technology, Guangzhou, Guangdong, China; 2 National Engineering Research Center for Tissue Restoration and Reconstruction, Guangzhou, Guangdong, China; University of Massachusetts, United States of America

## Abstract

Real-time quantitative reverse transcription-polymerase chain reaction (qPCR) is an efficient and accurate method to detect and compare patterns of gene expression. The reliability of qPCR is highly dependent on the selection of appropriate reference genes used for normalization. By analyzing 16 potential candidates of reference genes (GAPDH, Actb, 18 s, PGK1, Hprt, Tbp, Rpl5, B2M, Gusb, Ppia, UBC, Sdha, Eef1a1, H2afz, Tkt and Ldha) through geNorm, we identified Ppia, Tbp, Hprt and Eef1a1 as the most stable reference genes while UBC, B2M, Gusb as the least stable ones during the chondrocyte differentiation of ATDC5 cells. Considering the low expression of Eef1a1 and Tbp would cause divergent results for they failed to provide accurate normalization for RNA extraction and reverse transcription efficiency, we recommended the use of Ppia and Hprt as the most suitable genes to normalize qPCR. In addition, although GAPDH, Actb and 18 s were usually adopted in most of studies using ATDC5 cells, they were found unstable and then were not ideal reference genes for qPCR assay in ATDC5 cells chondrocyte differentiation. Also, we further confirmed that the Ppia and Hprt worked well during chondrocyte differentiation of mouse mesenchymal cells.

## Introduction

ATDC5 cells, which represent progenitor cells for chondroblasts, could easily form cell aggregates when growing in a medium supplemented with insulin and exhibit the entire spectrum of endochondral bone development [1], [2]. Therefore, this cell line is a useful *in vitro* model for deciphering the molecular mechanism of chondrogenesis, hypertrophy and endochondral ossification [3]–[5]. It is crucial to identify the expression profiles of specific genes during the process, which would bring important insight into understanding of the underlying molecular events.

As a powerful technique to rapidly quantify gene transcripts, real-time quantitative reverse transcription-polymerase chain reaction (qPCR) will undoubtedly play a pivotal role in deciphering the cellular and molecular properties of chondrocyte differentiation [6]. To obtain accurate results, suitable reference genes are employed to normalize cell number, RNA extraction and reverse transcription efficiency differences. Single reference gene, such as GAPDH, Actb and 18 s rRNA, has been generally used for normalization in more than 90% of studies [7], [8]. However, recently numerous reports showed that the expression levels of these widely used reference genes would vary in different tissues, cell types or even within the same tumor type after different biological treatments [9]. Consequently they were not suitable as internal control genes [10], [11]. Additionally, our previous study on chondrogenesis of ATDC5 cells found that the data was always in divergence or even in contradiction when using GAPDH and Actb as reference genes. Obviously, one or both of these two housekeeping genes was unsuitable for qPCR normalization under ATDC5 chondrocyte differentiation condition. Current consensus is that ideal and universal reference genes for all cell types and experiment conditions do not exist [8]. The mean expression of a group of reference genes with independent cellular functions is recommended due to its more accurate normalization [12]. Nevertheless, it may be impractical to measure the expression of multiple reference genes when only limited cDNA is available. Thus, the selection of suitable housekeeping gene for specific study is a prerequisite for qPCR assay to obtain reliable data.

In this study, geNorm^PLUS^ [8], [13] was used for selecting appropriate reference genes. The chosen of candidates were based on two principles: a) the reference genes have been widely used and confirmed stable in some cases; b) the chosen reference genes should perform different functions in cells. Besides GAPDH [14], [15], Actb [16], [17] and 18 s rRNA [18], [19], which were commonly used for qPCR normalization, we also chose other genes, which have been reported stable and could participate in different cell functions (briefly described in table 1). We aimed to find out the suitable reference genes for qPCR analysis during chondrocyte differentiation of ATDC5 cells.

**Table 1:** The reference genes chose in this study.

Abbr.	Name	Function	Reference
**GAPDH**	glyceraldehyde-3-phosphate dehydrogenase	glycolysis and gluconeogenesis	[14], [15], [23], [25]
**Actb**	actin, beta	cytoskeleton	[16], [17]
**18 s**	18Sribosomal RNA	translation	[18], [19]
**PGK1**	phosphoglycerate kinase 1	catalyzes the formation of ATP in Glycolysis	[23]
**Hprt**	hypoxanthine guanine phosphoribosyl transferase	purine synthesis	[24], [26]–[28]
**Tbp**	TATA box binding protein	transcription	[29]
**Rpl5**	ribosomal protein L5	component of the 60S subunit of ribosome	[24]
**B2M**	beta-2 microglobulin	antigen presentation	[8]
**Gusb**	glucuronidase, beta	hydrolysisof mucopolysaccharides	[29]
**Ppia**	peptidylproly lisomerase A	protein folding	[24], [27]
**UBC**	ubiquitin C	protein degradation	[27]
**Sdha**	succinate dehydrogenase complex, subunit A	citric acid cycle and respiratory chain	[23]
**Eef1a1**	eukaryotic translation elongation factor 1 alpha 1	translation biosynthesisand elongation	[26]
**H2afz**	H2A histone family, member Z	chromosome organization	[23], [27]
**Tkt**	transketolase	pentose phosphate pathway	[30]
**Ldha**	lactate dehydrogenase A	anaerobic glycolysis	[24]

## Materials and Methods

### Cell culture

The ATDC5 cell line was bought from ABGENT (San Diego, USA) and cultured in growth medium (GM) containing 1∶1 mixture of Dulbecco's modified Eagle's medium and Ham's F-12 medium supplemented with 5% fetal bovine serum (Invitrogen), 10 µg/ml human transferrin and 3×10^−8^ M sodium selenite (Sigma) in culture flasks at 37°C under 5% CO_2_. Mouse mesenchymal stem cells (mMSCs) was bought from ATCC (CRL-12424, USA) and cultured in growth medium containing Dulbecco's modified Eagle's medium supplemented with 10% fetal bovine serum (Invitrogen) in culture flasks at 37°C under 5% CO_2_.

### Chondrocyte Differentiation of cells

ATDC5 cells were cultured in 24-well plates with GM. After confluence, two-thirds of the medium was changed to insulin medium (IM) or TGF medium (CM), and the time was marked as 0 d. The ingredients of the above-mentioned medium were shown as follows: IM – growth medium supplemented with 10 µg/ml bovine insulin (Sigma); CM – high-glucose Dulbecco's modified Eagle medium (H-DMEM) (Gibco) with supplement of 10 ng/ml recombinant human transforming growth factor-β3 (TGF-β3) (Peprotech), 100 nM dexamethasone (Sigma), 50 µg/ml ascorbic acid 2-phosphate (Sigma), 1 mM sodium pyruvate (Amersco), 40 µg/ml proline (Biosharp) and ITS+ premix (BD; final concentrations: 6.25 µg/ml bovine insulin, 6.25 µg/ml transferrin, 6.25 µg/ml selenous acid, 5.33 µg/ml linoleic acid and 1.25 mg/ml bovine serum albumin).

mMSCs were harvested after confluence, and then cultured in 24-well plates with CM for 1, 7, 14 and 21 days. The number of cells in each well was 4×10^4^.

In all groups, medium was changed every 2 days.

### Real-time PCR analysis

Total RNA was isolated using TRIzol Reagent (Invitrogen) according to the manufacturer's protocol. The RNA concentration was determined using a NanoDrop2000 spectrophotometer (Thermo Scientific) and reverse transcription reactions were performed from 500 ng of total RNA using a First cDNA synthesis Kit (Fermentas). Real-time PCR reactions for 16 genes were performed using the SYBR green system (Invitrogen).

Primer sequences were listed in Table 2. Real-time PCR reactions were performed using the Chromo 4 Real time PCR system (Bio-rad). Samples were held at 95°C for 2 min, followed by 40 amplification cycles consisting of a denaturation step at 95°C for 15 s, and an annealing & extension step at 60°C for 1 min.

**Table 2:** Primers used in this study.

Primer	Forward sequence	Reverse sequence	Amplicon	A©ccess No.	Reference
**GAPDH**	TGACGTGCCGCCTGGAGAAA	AGTGTAGCCCAAGATGCCCTTCAG	98	NM_008084.2	[27]
**Actb**	TGACAGGATGCAGAAGGAGA	GCTGGAAGGTGGACAGTGAG	131	NM_007393.3	[31]
**18 s**	ATGCGGCGGCGTTATTCC	GCTATCAATCTGTCAATCCTGTCC	203	NG_032038.1	[32]
**PGK1**	CTGACTTTGGACAAGCTGGACG	GCAGCCTTGATCCTTTGGTTG	110	NM_008828.2	[23]
**Hprt**	CTGGTGAAAAGGACCTCTCGAA	CTGAAGTACTCATTATAGTCAAGGGCAT	110	NM_013556.2	[27]
**Tbp**	GAAGAACAATCCAGACTAGCAGCA	CCTTATAGGGAACTTCACATCACAG	129	NM_013684.3	[23]
**Rpl5**	GGAAGCACATCATGGGTCAGA	TACGCATCTTCATCTTCCTCCATT	70	NM_016980.2	[24]
**B2M**	CCGCCTCACATTGAAATCCA	TCGATCCCAGTAGACGGTCTTG	198	NM_009735.3	[23]
**Gusb**	GGCTGGTGACCTACTGGATTT	TTGGCACTGGGAACCTGAAGT	133	NM_010368.1	[23]
**Ppia**	CGCGTCTCCTTCGAGCTGTTTG	TGTAAAGTCACCACCCTGGCACAT	150	NM_008907.1	[27]
**UBC**	GAGCCCAGTGTTACCACCAAG	CATCACACCCAAGAACAAGCA	104	NM_019639.4	[27]
**Sdha**	GCTCCTGCCTCTGTGGTTGA	AGCAACACCGATGAGCCTG	136	NM_023281.1	[23]
**Eef1a1**	GCGGAGTTGAGGCTGCTGGAGA	AGACTCGGGCCATTGTTTGTCTG	110	NM_010106.2	[27]
**H2afz**	GCGCAGCCATCCTGGAGTA	CCGATCAGCGATTTGTGGA	196	NM_016750.2	[27]
**Tkt**	GACAGTGCCCTTCTGCAGTACTT	CCATGCGAATCTGGTCGAA	65	NM_009388.5	[24]
**Ldha**	ATCCCATTTCCACCATGATT	ACTGCAGCTCCTTCTGGATT	183	NM_010699.2	[24]
**Col2**	AGGGCAACAGCAGGTTCACATAC	TGTCCACACCAAATTCCTGTTCA	171	NM_031163.3	[25]
**Col1**	ATGCCGCGACCTCAAGATG	TGAGGCACAGACGGCTGAGTA	153	NM_007742.3	[25]
**Sox9**	GCTGGAAGTCGGAGAGCCGAGA	AGAGAACGAAACCGGGGCCAC	137	NM_011448.4	This study
**ColX**	TTCTGCTGCTAATGTTCTTGACC	GGGATGAAGTATTGTGTCTTGGG	115	NM_009925.4	[33]

For qPCR reactions, sample maximization strategies were used, i.e. all the samples were measured in the same run for a given reference gene [13].

### Statistical analysis

The data of qPCR assay for the set of 16 reference genes was analyzed with qBase^PLUS^ software (biogazelle). The detail of the algorithm for gene stability analysis can be found in the author's paper at Genome biology in 2002 [8]. Experiments were repeated with n = 3 biological replicates and the results were represented as the mean ± standard deviation. Repetitive ANOVA and Tukey's multiple comparison tests were used to determine statistical significance (P<0.05) between groups.

## Results

### Stability analysis of 16 reference genes

**Figure 1:**
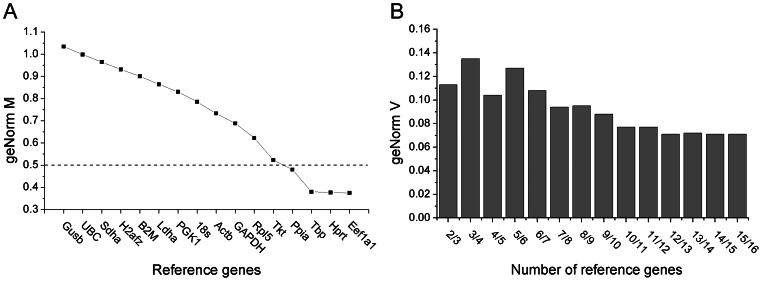
The values of geNorm M (A) and geNorm V (B) of 16 reference genes. (A) The genes with lower geNorm M values were considered more stable and the gene with M value below 0.5 was accepted as appropriate reference genes. Thus, Ppia, Tbp, Hprt and Eef1a1 were acceptable reference genes in this study. (B) The optimal number of reference genes required for normalization was determined by pairwise variation (geNorm V value of n/n+1) and a value below 0.15 indicated the minimum number (n) of genes. In this study, a combination of two reference genes was sufficient for normalization since V_2/3_ was 0.113.

**Figure 2:**
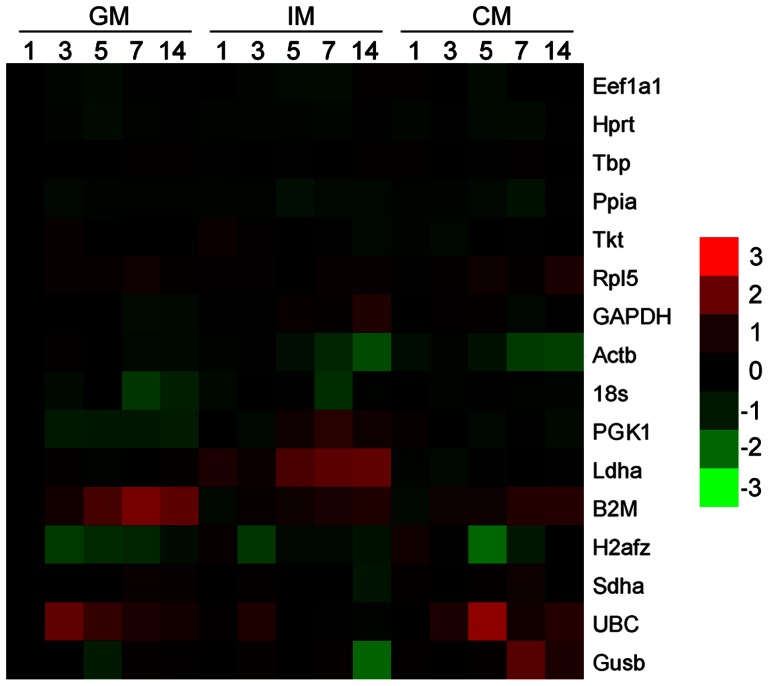
Heatmap for individual reference genes expression. The relative gene expression levels of all samples were depicted in color-code. The threshold cycle value of each sample was normalized against that of all the reference genes. Expression levels of all samples were normalized against those of 1 d GM group respectively and log_2_ transformed. Sixteen reference genes were sorted from up to down by their geNorm M value. Red color indicated that the expression level of gene was up-regulated compared to that of 1 d of GM group (the brighter the higher), whereas green color indicated vice versa (the brighter the lower).

To determine stable reference genes for *in vitro* chondrogenesis of ATDC5 cells, cells cultured in GM, IM and CM were analyzed at 1, 3, 5, 7 and 14 d. All the data of qPCR from different groups at various time points was inputted to geNorm following the instruction of the software. Then the final results were provided by geNorm as shown in Fig. 1. Also, the expression level of each reference gene at various time points was illustrated in Fig. 2. According to the geNorm manual, the genes with lower geNorm M value are considered more stable and the value 0.5 forms the division between stable and unstable reference genes. Thus, Ppia, Tbp, Hprt, and Eef1a1 were proved as stable genes (Fig. 1A). Heatmap shown in Fig. 2 further confirmed that the expression level of Eef1a1, Tbp, Hprt, and Ppia were relatively constant while genes with higher geNorm M value expressed less stably. Also, from Fig. 2 we can see that all reference genes were not co-regulated, i.e. the 16 genes were regulated randomly along time line in different culture medium. For example, Actb was down-regulated in all culture medium along the time line, while GAPDH was up-regulated in IM at all time points and in GM, CM before 7 d.

Pairwise variation (shown as geNorm V) is used to determine the optimal number of reference genes required for normalization. The geNorm V value below 0.15 suggests a minimum required number of reference genes [8] for normalization. Thus, in this study a combination of two reference genes was sufficient for normalization as indicated in Fig. 1B.

### Expression profiles of target genes normalized against various reference genes in ATDC5 cells

**Figure 3:**
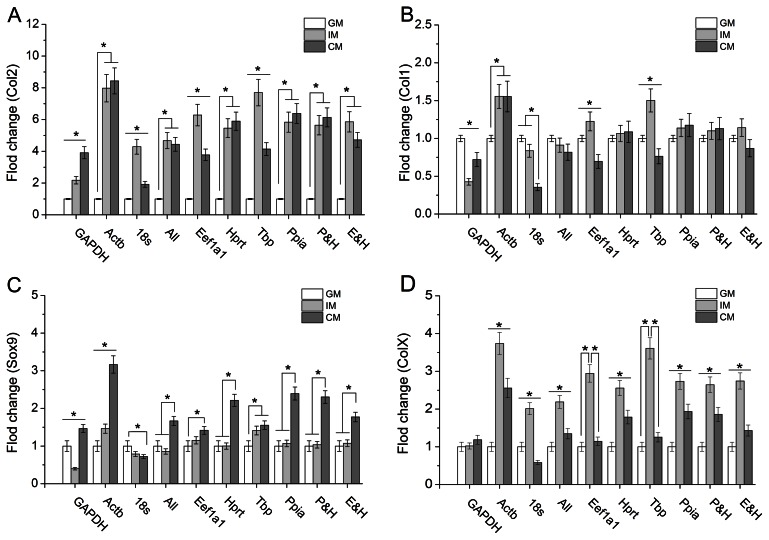
Relative expression of various genes normalized against different reference genes in ATDC5 cells. (A) Col2; (B) Col1; (C) Sox9; (D) ColX. *P<0.05 represented the significant effects among different culture medium.

To exhibit the influence of reference genes on the expression profiles of target genes, cells cultured in GM, IM and CM were harvested at day 7 followed by qPCR assay. Then the data of fold change for chondrogenesis, hypertrophy and endochondral ossification related genes – Collagen type I, Collagen type II, Transcription factor SOX-9 and Collagen type X (Col1, Col2, Sox9 and ColX) was obtained using ΔΔCt methods. In the calculation, various reference genes were employed including widely used genes (designated as GAPDH, Actb and 18 s), the stable reference genes demonstrated in this study (designated as Eef1a1, Hprt, Tbp and Ppia), the combination of Eef1a1 & Hprt and Ppia & Hprt (designated as E&H and P&H, the geometric mean of two reference genes Ct value) as well as the combination of sixteen reference genes (designated as “All”, the geometric mean of all reference genes Ct value). As shown in Fig. 3, the expression profiles of Col1, Col2, Sox9 and ColX in Ppia, Hprt, E&H and P&H groups indicated similar to those in “All” group while the trends of these target genes expression in GAPDH, Actb, 18 s, Eef1a1 and Tbp groups exhibited inconsistent with those in “All” group. Compared with “All” group in which there was no significance between CM and IM, the expression level of Col2 in GAPDH group suggested that CM was more beneficial to Col2 synthesis than IM while contrary results showed up in 18 s, Eef1a1 and Tbp group (Fig. 3A). It was shown that IM promoted Col1 expression in Actb, Eef1a1 and Tbp groups but inhibited it in GAPDH group (Fig. 3B). For Sox9 and ColX expression, similar results could be observed (Fig. 3C and Fig. 3D).

### Comparison of mRNA expression levels among 16 reference genes in ATDC5 cells

**Figure 4:**
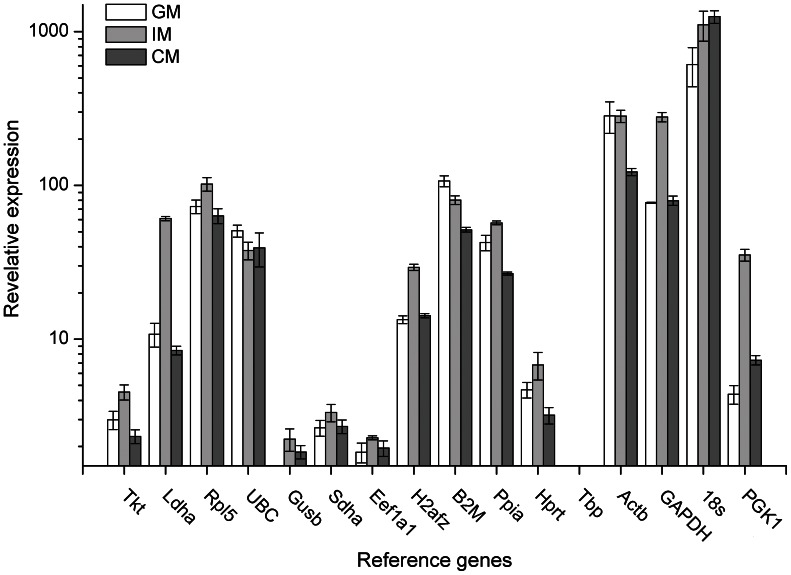
Relative expression of sixteen candidate reference genes in ATDC5 cells. Cells were cultured in different medium for 7 d. The data were calculated using ΔCt methods with normalization against Tbp. Error bars represented the standard error of 3 biological replicates. Note that the Y axis was on a log_10_ scale.

ATDC5 cells cultured in different medium were harvested at day 7 followed by RNA extraction and qPCR assay. The Ct value of each reference gene was normalized against Tbp, which had the lowest value. As shown in Fig. 4, the quantitative data of all the samples could be categorized into three groups by magnitude: 1 to 10 (Tbp, Eef1a1, Gusb, Sdha, Tkt, Hprt and PGK1), 10 to 100 (Ldha, Rpl5, UBC, H2afz, B2M, Ppia and GAPDH) and above 100 (Actb and 18 s). Knowing the expression levels of the reference gene candidates would help us choose proper reference genes with similar transcription level to the target genes.

### Confirmation the suitability of Ppia and Hprt during chondrocyte differentiation of mMSCs

**Figure 5:**
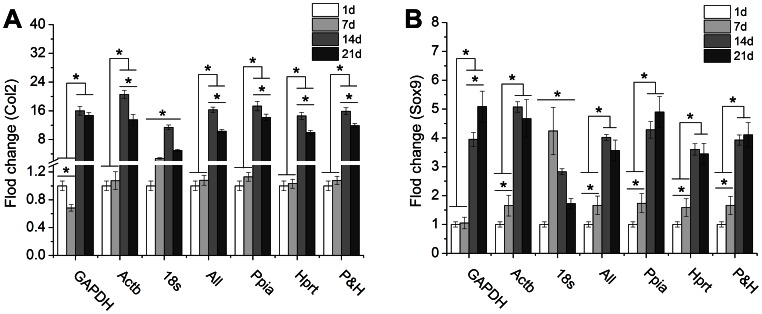
Relative expression of cartilage-specific genes normalized against different reference genes in mMSCs. Col2; (B) Sox9. *P<0.05 represented the significant effects among different culture time.

In order to further verify whether Ppia and Hprt could work well in chondrocyte differentiation of mMSCs, mMSCs cultured in CM were harvested at day 1, 7, 14 and 21 followed by RNA extraction and qPCR assay. Then the data of fold change for Col2 and Sox9 was obtained using ΔΔCt methods. Various reference genes used in this study were employed. As shown in Fig. 5, the expression profiles of Col2 and Sox9 in Ppia, Hprt, and P&H groups were similar to those in “All” group, which demonstrated that these two reference genes picked out in this study were also stable and suitable as qPCR reference genes during chondrocyte differentiation of mMSCs. The trend of Col2 (Fig. 5A) or Sox9 (Fig. 5B) expression along time line in GAPDH and 18 s group was quite different from that in “All” group.

## Discussion

Some algorithms such as geNorm [8], Norm Finder [20] and Best Keeper [21] have been developed to select the optimal reference genes for various experimental conditions. Thereinto, geNorm developed by Vandesompele et al. [8] in 2002 was well acknowledged and usually showed similar results to Norm Finder and Best Keeper [22]–[24]. In this study, geNorm^PLUS^ [8], [13] was used for reference gene selection. It was based on the principle that the expression of two ideal reference genes should be identical in all samples, regardless of the experimental condition or cell type. According to this principle, the unsuitable genes would be eliminated from all existing candidates by calculating their geNorm M value. Then the remaining genes would be screened until one or several optimal reference genes could be confirmed under a given experimental condition.

At the beginning of the study, reference genes taking part in different functions were chosen because these genes may not be co-regulated simultaneously. Otherwise, genes with the same function would probably be regulated at the same time, which could lead to misjudgment [8]. Moreover, to increase the reliability of the results, we adopted three different cell media in this study. IM medium was used in most studies with ATDC5 cells for chondrocyte differentiation [1], [2], [15]. Also, Tare et al. [3] reported that CM, commonly used for chondrogenesis of MSCs and other stem cells, was effective for chondrocyte differentiation of ATDC5. GM was used for cultivation of ADTC5 cells. Thus, the results would be more reliable with various experimental conditions. Following the rule of geNorm that reference gene with geNorm M below 0.5 was regarded as stable genes, we screened Ppia, Tbp, Hprt and Eef1a1 out. Although geNorm^PLUS^ can recognize the most stable reference gene from the given reference gene candidates, the software still recommended the use of two or more reference genes instead of one for accurate normalization to exclude potential instability [8], [13]. The pairwise variation coefficient (shown as geNorm V) was used to determine the optimal number of reference genes required for normalization. According to the instruction of geNorm, n would be regarded as optimal number of reference gene, when the value of V_n/n+1_ drops below 0.15 and genes with n lowest values of geNorm M would be the final choice. As shown in Fig. 1B, since V_2/3_ was less than 0.15, it suggested that the optimal number of reference genes in this experimental situation was 2 (Eef1a1 and Hprt, which have the lowest geNorm M value). Considering the low expression levels of Eef1a1 and Tbp (Fig. 4), we excluded Eef1a1 and Tbp and recalculated the remaining genes. We found that Ppia and Hprt as the most stable genes and their geNorm V value of 2/3 was below 0.15 (data not shown).

In addition, we compared the qPCR data normalized against the following reference genes respectively: GAPDH, Actb,18 s, Eef1a1, Hprt, Tbp, Ppia, E&H (Eef1a1 and Hprt), P&H (Ppia and Hprt) and “All” (16 reference genes). It was recognized that the more reference genes were used in qPCR data process, the higher accuracy could be obtained. The respective results from Ppia and Hprt were similar to those from “All”, i.e. these two reference genes were more suitable than others and could be used as a single reference gene in our study. Furthermore, the E&H and P&H, recommended by geNorm (according to the value of geNorm V) showed similar results to “All”, demonstrating the efficiency of this algorithm. Although Eef1a1 and Tbp were demonstrated as stable genes by means of geNorm, the data normalized against these two genes was inconsistent with that normalized against 16 reference genes. This might ascribe to the huge difference of the expression level between target genes and reference genes, for the low expression of reference genes failed to provide accurate normalization for RNA extraction and reverse transcription efficiency of high expression target genes. As mentioned by other researchers, target genes and reference genes should have comparable expression level [12]. Additionally, the widely used GAPDH, Actb and 18 s, showed significant divergence from each other. Hence, they should not be appropriate reference genes for ATDC5 cells chondrocyte differentiation. Also, in the study of mMSCs chondrocyte differentiation, similar expression profiles of Col2 and Sox9 in Ppia, Hprt, and P&H groups to those in “All” group demonstrated that these two reference genes Ppia and Hprt picked out in this study were also stable and suitable as qPCR reference genes during chondrocyte differentiation of mMSCs.

In conclusion, we suggest the use of Ppia & Hprt when normalizing gene expression in chondrocyte differentiation of ATDC5 cells and mMSCs. Considering the economic principle and the results in the present study, we also recommend the sole use of Ppia or Hprt for normalization during chondrocyte differentiation. In addition, our results highlight the importance of choosing appropriate reference genes with comparative copies against target genes during qPCR normalization.
